# Flavonoids-Based Delivery Systems towards Cancer Therapies

**DOI:** 10.3390/bioengineering9050197

**Published:** 2022-05-02

**Authors:** Miguel Ferreira, Diana Costa, Ângela Sousa

**Affiliations:** CICS-UBI—Health Science Research Centre, University of Beira Interior, Av. Infante D. Henrique, 6200-506 Covilhã, Portugal; miguel.r.ferreira@ubi.pt

**Keywords:** anticancer activity, cervical cancer, delivery systems, flavonoids, HeLa cells, tumor inhibition

## Abstract

Cancer is the second leading cause of death worldwide. Cervical cancer, for instance, is considered a major scourge in low-income countries. Its development is mostly associated with the human papillomavirus persistent infection and despite the availability of preventive vaccines, they are only widely administered in more developed countries, thus leaving a large percentage of unvaccinated women highly susceptible to this type of cancer. Current treatments are based on invasive techniques, being far from effective. Therefore, the search for novel, advanced and personalized therapeutic approaches is imperative. Flavonoids belong to a group of natural polyphenolic compounds, well recognized for their great anticancer capacity, thus promising to be incorporated in cancer therapy protocols. However, their use is limited due to their low solubility, stability and bioavailability. To surpass these limitations, the encapsulation of flavonoids into delivery systems emerged as a valuable strategy to improve their stability and bioavailability. In this context, the aim of this review is to present the most reliable flavonoids-based delivery systems developed for anticancer therapies and the progress accomplished, with a special focus on cervical cancer therapy. The gathered information revealed the high therapeutic potential of flavonoids and highlights the relevance of delivery systems application, allowing a better understanding for future studies on effective cancer therapy.

## 1. Cervical Cancer

Cancer is currently one of the leading causes of death worldwide, estimated by the Global Cancer Observatory (GLOBOCAN) database that approximately 10 million people died from this disease in 2020 [1]. This illness is characterized by uncontrolled and abnormal cell growth due to the action of an initiated agent that causes abnormalities in the cell machinery or mutations in the DNA, which after cancer progression can trigger metastases in other parts of the body.

Cervical cancer is the fourth type of cancer with the highest incidence among women worldwide (6.5%). In less developed countries, it is the second type of cancer with the highest incidence as well as the second leading cause of death from cancer in women, 21.1% and 22.1%, respectively, being surpassed only by breast cancer [2,3]. The main factor in the development of cervical cancer is attributed to the human papilloma virus (HPV), accounting for 79% to 100% of cases, and of these, about 70% of cases are related to two high-risk subtypes, namely HPV-16 and HPV-18 [3,4].

The HPV genome codes for eight genes that can be divided into two groups according to the period of infection where they are produced [5]. The early region consists of genes E1, E2, E4, E5, E6 and E7 that are involved in the processes of replication, regulation of transcription and oncogenesis [6]. The late region codes for the L1 and L2 genes which are responsible for the formation of the capsid that protects the viral genome [6].

After infection, E1 and E2 proteins start to be expressed in the undifferentiated epithelial cells of the basal layer that are infected, regulating viral replication and the expression of other initial viral proteins [7]. At this point, the viral replication cycle is totally dependent on the differentiation cycle of the infected cells, since the DNA of the virus only replicates when the DNA of the infected cells is replicated [6]. Once the basal infected cells differentiate, infected cells will start expressing two viral proteins, E6 and E7, which will act as stimulators of cell proliferation, prolong cell cycle progression and prevent apoptosis [8]. The E6 and E7 oncoproteins will directly interfere with the p53 and pRb tumor suppressor proteins, respectively [3]. In the course of infection, the oncoprotein E6 will induce p53 degradation via ubiquitin in the proteosome, thereby impairing the apoptosis induction of infected cells [7,8]. The accumulation of DNA damage, due to the action of oncoproteins E6 and E7, leads to the appearance of cervical lesions and the establishment of a persistent infection [7]. Lastly, in the most differentiated cells, there is also the production of virions that are released by the keratinocytes, located more superficially, as they die [7].

**Figure 1:**
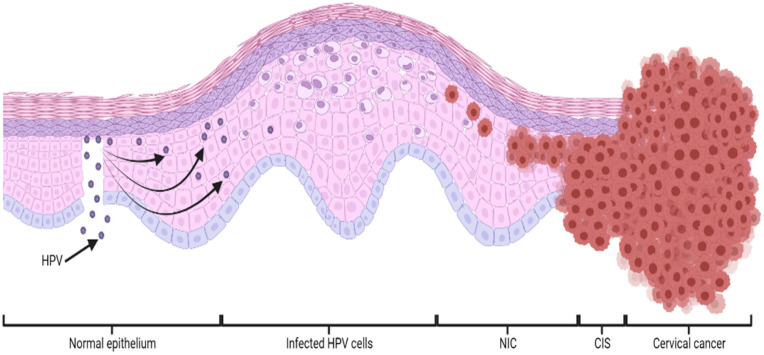
Schematic representation of HPV-mediated cervical epithelial basal cell infection over time. HPV (human papilloma virus); NIC (neoplasm); CIS (carcinoma in situ).

Therefore, HPV infection is transmitted through skin-to-skin contact, due to microlesions in the basal layer of the epithelium, causing neoplasms (NIC), carcinoma in situ (CIS) and ultimately leading to cervical cancer, as schematized in Figure 1 [6].

Although vaccines currently exist to prevent the main types of HPV, in countries where healthcare is insufficient and where these vaccines are not widely administered, HPV persistent infections and progression to cervical cancer are very common and the mortality rate is much higher compared to developed countries [2,3]. Therefore, it is of special interest to develop therapies to treat people who develop HPV-induced cancer. For this purpose, different approaches have been studied, namely through therapeutic DNA or RNA vaccines, natural polyphenolic compounds such as flavonoids and other chemical species such as alkaloids, polysaccharides, protein-based entities and plant extracts [8]. The use of natural compounds displaying low toxicity, high anticancer potential and low cost are currently being studied for the treatment of cervical cancer and other cancers. In this context, flavonoids appeared as the main types of compounds under study, being reported as having a significant viability reduction in in vitro assays caused by, among others, inhibition of oncoprotein E6 leading to an increase in p53 and post-induction of apoptosis [7,9].

## 2. Flavonoids

**Figure 2:**
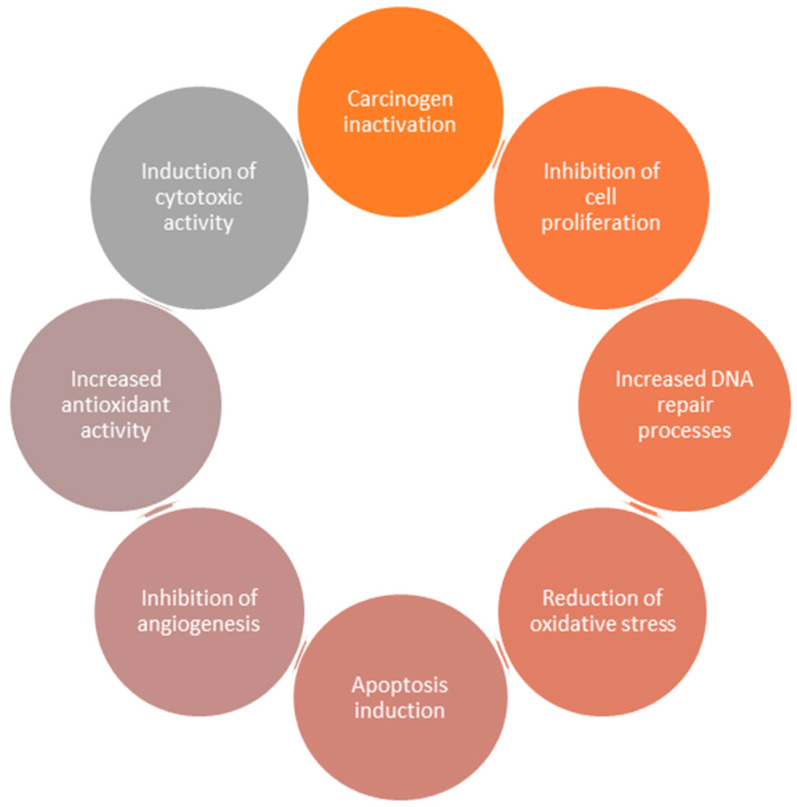
Schematic representation of the main mechanisms responsible for the anticancer potential of flavonoids.

Flavonoids are a group of phenolic compounds present in plants, fruits and vegetables characterized by having a carbon skeleton base of C6-C3-C6. This group of compounds is widely consumed in the human diet and has great scientific interest, having already been reported as antidiabetic [10], antioxidant [11], anti-inflammatory [12], antimicrobial [13], antiviral [13] and anticancer agents [14]. Nowadays, the main focus is based on the use of flavonoids in anticancer therapies, with the use of epigallocatechin-3-gallate, quercetin, genistein, luteolin, apigenin, silibinin, naringenin and kaempferol being reported [15,16]. These flavonoids exhibited significant anticancer capacity, namely through mechanisms such as carcinogen inactivation, induction of cytotoxic activity, increased antioxidant activity, inhibition of angiogenesis, apoptosis induction, reduction of oxidative stress, increased DNA repair processes and inhibition of cell proliferation [15,16]. All these mechanisms are summarized in Figure 2.

Although all mechanisms that contribute to the anticancer capacity of flavonoids have been reported, the pathway of each one is not yet fully understood. Therefore, studies are still carried out to fully unravel the mechanisms of action of flavonoids, in an attempt to improve their effects and decrease their limitations. For instance, the reduction of reactive oxygen species (ROS) and the induction of apoptosis through death receptors are considered the two main mechanisms of action, present in the vast majority of flavonoids [17].

**Figure 3:**
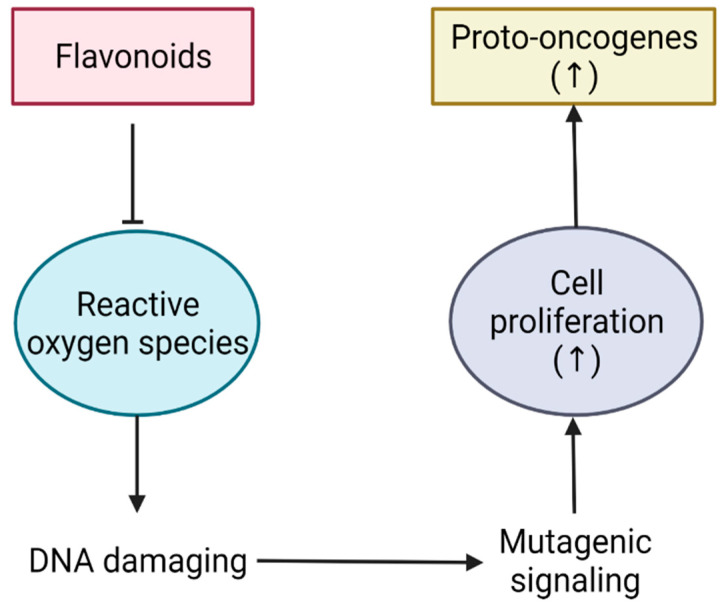
Scheme illustrating the flavonoid action on the reactive oxygen species (ROS) pathway.

The reduction of ROS is one of the main mechanisms of action displayed by flavonoids, being responsible for the inhibition of cell proliferation and a resulting decrease in tumor growth [17]. ROS are largely responsible for causing irreversible DNA damage leading to the creation of cancer cells and their uncontrolled growth. Beyond that, they are also responsible for the production of proto-oncogenes that can induce even greater cell growth [17,18]. The action of flavonoids allows a decrease in ROS and, in turn, a decrease in cell proliferation and proto-oncogenes, thus reducing the risk of progression to more severe stages of cancer (Figure 3) [18].

**Figure 4:**
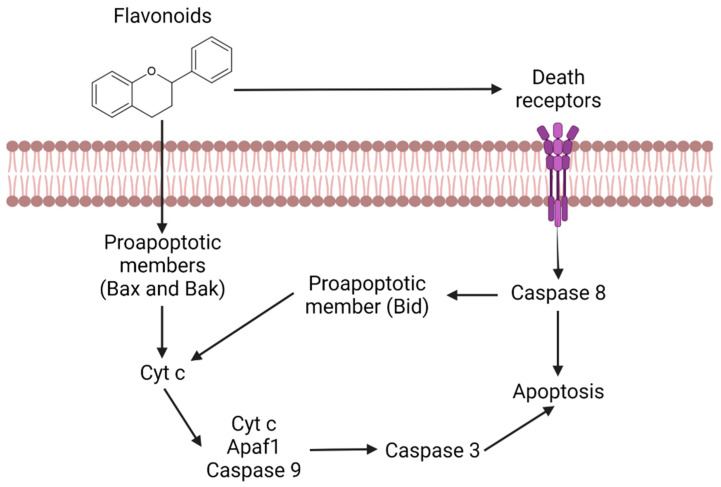
Representation of the apoptotic pathway mediated by flavonoids.

The apoptosis induction is another of the main mechanisms of action, being responsible for the activation of death receptors as well as the activation of Bax and Bak proapoptotic members (Figure 4) [17]. Bax, Bak and Bid are apoptosis regulator proteins that have the ability to release cytochrome c in the mitochondria [17]. This release induces caspase activation, namely caspase 9 and 3, leading cells to apoptosis [18].

On the other hand, flavonoids can activate death receptors that will stimulate caspase 8 as well as the Bid proapoptotic member, which in turn allows the release of cytochrome c [18]. Thereby, in any of these pathways, the cells will always enter apoptosis, therefore promoting tumor reduction.

Depending on the type of flavonoid, its action can be very different, with some flavonoids showing differences in antioxidant power, ability to reduce ROS, and inducing apoptosis, among others [19].

Flavonoids also have another mechanism of action in cervical cancer, namely through the inhibition of oncoprotein E6 [7]. As mentioned earlier, inhibition of this oncoprotein results in an increase in p53 levels, thus leading to a subsequent increase in apoptosis [7]. The presence of this mechanism represents an improvement in flavonoid action in relation to other types of cancer, boosting their applications.

Furthermore, the use of flavonoids as adjuvants to chemotherapeutic agents, such as doxorubicin or paclitaxel, has also been the subject of intensive studies [20,21,22,23]. Their application in this type of therapy promoted intracellular accumulation of the drug in cancer cells and a significant decrease in the proliferation/growth of these cells. Additionally, they contributed to reduced toxicity in healthy cells alleviating side effects. The combined action of flavonoids and anticancer drugs, thus, overcomes one of the main limitations of chemotherapy and potentiates the anticancer effect of the drug at a lower concentration [20,21,22,23].

### 2.1. Flavonoids Subgroups

**Figure 5:**
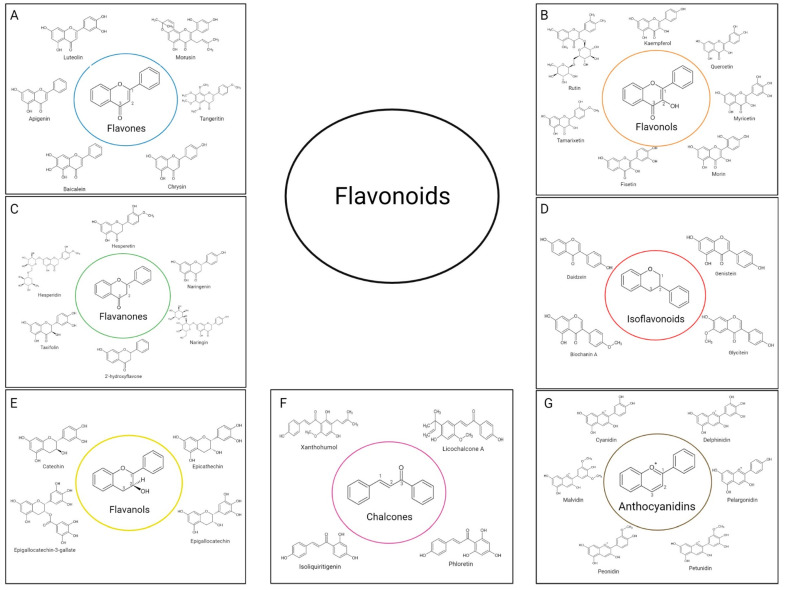
Classification of flavonoids: (**A**) flavones, (**B**) flavonols, (**C**) flavanones, (**D**) isoflavonoids, (**E**) flavanols, (**F**) chalcones, (**G**) anthocyanidins.

Flavonoids are a class of polyphenols that can be classified according to their biosynthetic origin, as well as according to the binding site of the aromatic rings and the degree of oxidation and saturation of the carbons present between the rings [24]. Currently, flavonoids can be divided into several subgroups. The most important subgroups are summarized in the diagram of Figure 5, being the characteristics and main assets of each subgroup described in the points below.

#### 2.1.1. Flavones

Flavones are characterized by a double bond between carbons represented by numbers 1 and 2, and a ketone group on the carbon represented by number 3 (Figure 5A) [25]. The most scientifically relevant flavones are luteolin, morusin, tangeritin, chrysin, baicalein and apigenin, also represented in Figure 5A [25]. In nature, flavones are usually found in glycosylated form and can be extracted from fruits, leaves and flowers, with parsley, red pepper, mint, celery, chamomile, orange, honey and ginkgo biloba being the main sources of this group [26]. Chemically, flavones are used to regulate the interactions of insects and microorganisms with plants, protecting them. In addition, they are also part of the group of non-essential nutrients, being widely used in the food industry for this reason [17].

In humans, flavones have several benefits such as the ability to decrease the production of ROS through the inhibition of the xanthine oxidase enzyme, thus inhibiting the efflux pumps and leading to the induction of apoptosis. Furthermore, flavones also interact with estrogen receptors, preventing them from losing their shape, and therefore, binding to cancerous co-activators [19,24].

#### 2.1.2. Flavonols

Flavonols differ from flavones essentially in a higher oxidation state with the base skeleton of this group containing an -OH group on carbon represented by the number 2 in Figure 5B. As the most represented group in nature, flavonols can be extracted from fruits and vegetables such as bananas, apples, onions, broccoli and grapes, but also in red wine and teas such as black and green tea [24]. Quercetin is the main flavonol and the most relevant in scientific studies and therapeutic actions but there are also other flavonols with high scientific interest such as kaempferol, rutin, myricetin, morin, fisetin and tamarixetin (Figure 5B).

In nature, flavonols appear to play an important role in plant growth and development, as well as resistance to UV radiation and insects [17]. In human health, flavonols are relevant due to their antioxidant, antimicrobial, anti-inflammatory, cardiovascular and anticancer activities. Quercetin is the most clinically studied flavonol and its anticancer role has already been proved, namely the inhibitory effect on the growth of cancer cells. It prevents metastases by inhibiting the secretion of matrix metalloproteinases (MMPs), inhibiting epigenetic changes, interrupting progression in the cell cycle and promoting further apoptosis of cancer cells, preventing oxidative stress and inhibiting angiogenesis [9,17,20,27,28].

#### 2.1.3. Flavanones

Flavanones, characterized by having saturated and deoxidized carbons represented by numbers 1 and 2 in Figure 5C are the first product of the flavonoid biosynthesis pathway and the most reactive flavonoid group. Its presence in nature is mainly in the skin of fruits, in seeds, bark and flowers of most plants [24]. Hesperidin, hesperetin, naringenin, naringin, 2′-hydroxyflavone and taxifolin are the main flavanones (Figure 5C). Like flavonols, flavanones also have the ability to inhibit the expression of MMPs, thus decreasing their invasiveness of tissues and the appearance of metastases [29]. In addition, flavanones also have an antioxidant and anti-inflammatory action [25].

#### 2.1.4. Isoflavonoids

Isoflavonoids are present in plants of the *Fabaceae* family, namely soybeans, red cover, and chickpea, where they appear as secondary metabolites. They play an important role in protecting the plant against attacks from pathogenic microorganisms. Structurally, this group of flavonoids is distinguished from others due to the phenol group attached to the carbon, shown with number 2, instead of linked to the carbon shown in number 1 in Figure 5D [19,30]. Isoflavonoids can appear in their glycosylated and aglycosylated form (the aglycone forms). The main isoflavonoids can be found also in Figure 5D, namely Daidzein, Genistein, Glycitein and Biochanin A, which can also appear in glycosylated forms, namely in the form B-glycisudem, 6”-O-malonyl-glycoside and 6”-0-acetyl-glycoside [31].

In humans, their role in therapeutic uses has been the subject of extensive study and it was verified that isoflavonoids are useful in the prevention of diseases such as osteoporosis in women, diabetes, Kawasaki syndrome, Alzheimer’s and heart disease. Furthermore, isoflavonoids also display antioxidant capacity due to their ability to donate hydrogen atoms from the groups attached to the benzylic ring, as well as their ability to activate and express the antioxidant enzymes catalase, glutathione and superoxide dismutase [19,30]. Beyond these features, their most important role in medical therapy is related to anticancer capacity. Due to their structural similarities with estrogens, isoflavones have also the ability to bind to estrogen receptors, thus competing with them and decreasing cancer-related estrogens. In this way, isoflavones have been widely used in therapies against breast cancer, among other cancer types [30].

#### 2.1.5. Flavanols (Flavan-3-ols)

Flavanols, or Flavan-3-ols, are characterized by a structure with the carbons represented by numbers 1, 2 and 3 fully saturated, as well as a hydroxyl group on the chiral carbon represented by number 2 (Figure 5E) [32]. The main compounds of this group are catechin, epicatechin, epigallocatechin and epigallocatechin-3-gallate, and due to their name, this group may also have the name catechins [32]. Flavanols are mainly present in tea leaves but can also be found in chocolate, cocoa and apples [32]. In humans, flavanols are described to have the ability to inhibit the activity of digestive enzymes such as α-amylase and α-glucosidase, which lead to a reduction in blood glucose levels. Furthermore, they have been reported to exert an effect on blood pressure, lowering it, on reducing the risk of cardiovascular disease. Additionally, flavanols display antioxidant, antidepressant and anti-obesity properties [19].

#### 2.1.6. Chalcones

Considering the chemical structure, chalcones are the most differentiated group of flavonoids due to the fact that carbons represented by numbers 1, 2 and 3 in Figure 5F do not form a third ring located between the two benzylic rings. In addition, chalcones also have a ketone group on the carbon represented with the number 3 in the same figure. This group of open-chain flavonoids can be easily found in plants of *Moraceae*, *Leguminosae* and *Compositae* families, being present in vegetables, fruits, grains, roots, flowers, teas and wines [33]. The main flavonoids in this subgroup used for therapeutic purposes are licochalcone A, phloretin, xanthohumol and isoliquiritigenin [25].

In nature, this group has the ability to defend plants, neutralizing ROS and thus preventing molecular damage and attacks by microorganisms [34]. Concerning humans, chalcones have been reported to have several properties for medicinal purposes, including antifungal, anti-inflammatory, antibacterial, antiviral, anticancer, and neuroprotective, among others. The ability to inhibit ROS is thought to be mainly responsible for all these therapeutic properties [34].

#### 2.1.7. Anthocyanidins

As the only group that has considerable aqueous solubility, anthocyanidins are characterized by an unsaturated ring between the two benzylic rings, forming a flavylium cation (Figure 5G) [35]. Anthocyanidins are abundantly found in pigments of leaves, flowers, vegetables and fruits, being responsible for the colors between the ranges of blue, red and purple. In addition to its pigmentary properties, the inhibition of ROS and the regulation of the physiological stage of tissues in plants are also a relevant part of their valences [36]. The main flavonoids present in this group are cyanidin, delphinidin, malvidin, pelargonidin, peonidin and petunidin (Figure 5G) [36]. In humans, anthocyanidins have neuroprotective, antioxidant, anti-diabetic, anti-obesity and anticancer capacities, being responsible for inhibiting cancerous growth [36].

### 2.2. Bioavailability

Despite the low toxicity displayed by all flavonoids, along with the great value in a therapeutic environment already mentioned, almost all groups present very low bioavailability. This parameter greatly varies from one compound to another. Isoflavones, flavanones, quercetin glucosides and flavanols exhibit the highest bioavailability among all flavonoid subgroups, although being equally low for clinical purposes [15,31]. With the exception of anthocyanidins that display significant aqueous solubility, the solubility in water is generally very low across all flavonoid groups; thus, their absorption is difficult by oral administration, therefore decreasing their bioavailability and therapeutic effect [35,37]. Furthermore, flavonoids have low stability, easy degradation in extremely acidic medium, low intestinal permeability and high metabolism making the bioavailability of considered flavonoids very low [10].

To overcome the bioavailability drawbacks, several approaches based on delivery systems are being developed and are focused on processes to increase intestinal absorption, improve stability or change the absorption site [38,39,40,41]. In addition, the use of delivery systems allows for improving flavonoid solubility, decreasing gastrointestinal degradation, improving absorption into the bloodstream, protecting against renal clearance and also protecting against secretion in the liver. In line with this, the study of flavonoid-based delivery systems acquired high scientific importance [42].

## 3. Delivery Systems

**Figure 6:**
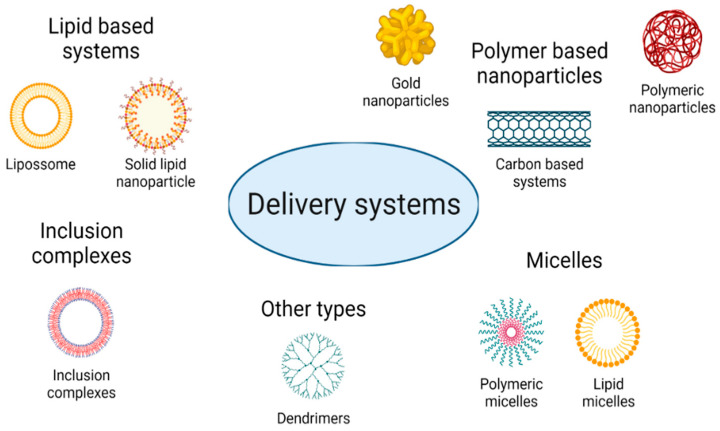
The main types of delivery systems explored for drug encapsulation.

Several types of flavonoid-based delivery systems are being developed for anticancer therapy, namely for cervical cancer. The consideration of these new forms of delivery systems allows for the use of fewer flavonoids as well as permits the use of ligands that efficiently target these systems to cancer cells, therefore reducing the risk of toxicity in healthy cells and improving their therapeutic effect. A variety of delivery systems for the encapsulation of these drugs can be explored, depending on the material considered and the properties exhibited by the drug. In this way, delivery systems can be very differentiated in nature, with the main groups summarized in Figure 6.

The characteristics of each type of system and the compounds that constitute them can be analyzed in the topics below. Table 1 summarizes the delivery systems and also indicates the ones already developed for cervical cancer therapies in in vitro (Hela cells) and in vivo assays (U14 cervical carcinoma).

### 3.1. Lipid-Based Delivery System

Delivery systems based on lipids include three large groups: liposomes, solid lipid-based nanoparticles and emulsions, and in each of these groups, there may still be divisions into subgroups [69].

#### 3.1.1. Liposomes

Liposomes are spherical vesicles typically made up of emulsifiers and a bioactive compound dissolved in an organic solvent. Liposomes are usually made of at least one lipid layer which allows them to be used very often for the encapsulation of both hydrophilic and hydrophobic drugs [43,70]. The constitution of liposomes essentially includes phospholipids and may also contain cholesterol or even a hydrophilic polymer such as polyethylene glycol (PEG). In this case, they can be called stealth liposomes, as the junction with this type of polymer prolongs the circulation time of liposomes increasing their efficiency. Regarding the formation of liposomes with phospholipids and cholesterol, their use guarantees structural and biological stability, thus giving rise to biocompatible transport systems displaying high efficiency of encapsulation for all types of flavonoids, which is always superior to 80% and in many cases greater than 95% [44]. In addition, liposome stability also reduces the ability of systems to form aggregates, be toxic for therapeutic application and increase the capability of controlled release of the encapsulated drugs. However, they still show some physical and chemical instability leading to aggregation issues over time, as well as some drug degradation over the storage period [44,45,71]. Nonetheless, the use of this type of system is also favoured by its size and polydispersity index (PdI) which vary between 100 and 200 nm and 0.1 and 0.25, respectively. These favourable characteristics confer these systems’ ability to pass through the pores present in blood capillaries more easily and accumulate in tumors, which usually feature a much higher number of pores in the surrounding blood capillaries [44,45].

In vitro and in vivo studies demonstrated a large number of benefits of liposome application, namely due to their high capacity to encapsulate flavonoids, leading to the increase in therapeutic effect and low toxicity. Viability studies in cervical cancer cell lines (HeLa) demonstrate that, by encapsulating flavonoids into liposomes, a lower concentration of flavonoids is needed to obtain 50% inhibition (IC_50_) in cellular viability, which can decrease from 200 µM in the case of free quercetin to concentrations around 100 µM after a 24 h incubation (Table 1). For increased incubation times, an IC_50_ can be obtained for concentrations of 14 µM using liposomes made of triglycerides, lecithin, PEG and folic acid [45,46]. Furthermore, in vivo studies show that quercetin-loaded liposomes made of PEG, cholesterol and soybean phosphatidyl-choline induce a reduction in tumor size of about three times compared to the administration of free quercetin [43,46]. Other liposomes constituted of soybean phosphatidylcholine and cholesterol show a tumor volume decrease of about 50% [43]. In the context of other cancers, the use of liposomes has also been studied and shows equally a high rate of encapsulation, low toxicity and high percentage of cell inhibition in in vitro studies [72,73,74]. Overall, a wide variety of emulsifiers have already been tested in anticancer therapies, such as lecithin or PEG derivatives. In some cases, chitosan coatings are explored in order to increase their bioavailability and stability in vivo, thus opening novel possibilities to improve the application of these systems in studies related to cervical cancer.

#### 3.1.2. Lipid-Based Nanoparticles

Another type of delivery system widely investigated is solid lipid-based nanoparticles, which are characterized by forming solid lipid systems at room temperature. They can be divided into solid lipid nanoparticles (SLNs) and nanostructured lipid carriers (NLCs). The main differentiating feature between SLNs and NLCs is the fact that SLNs form totally solid systems with a perfect crystal structure, while NLCs have a non-ideal crystal structure leading to the development of systems with a solid and a liquid zone, increasing drug loading and decreasing water content [75,76,77]. Compared to liposomes, SLNs makes it possible to avoid the need to use organic solvents, therefore reducing the cytotoxicity they present while maintaining a high capacity to encapsulate both hydrophobic and hydrophilic drugs [71]. Therefore, SLNs have high bioavailability, low cost/easy production on a large scale, and high capacity to maintain a controlled release [71]. In terms of constitution, both SLNs and NLCs are mainly composed of non-ionic surfactants such as Polysorbate 80^®^, Poloxamer 188, Tyloxa-pol and sometimes, made up of lecithin or phosphatidylcholine as their amphoteric nature improves the stability exhibited by the system [59]. In the case of NLCs, liquid lipids are used to make the liquid part of the system, such as oleic acid, olive oil, almond oil and cetiol^®^ [71]. Despite all their advantages and their high tolerance in in vitro and in vivo tests for being composed of natural compounds, the toxicity of surfactants and other excipients necessary for their production must be considered. In addition, there is still a risk of aggregation and recrystallization [69,78].

The application of SLNs and NCLs in in vitro and in vivo assays related to cervical cancer is not yet fully tested. However, taking into account studies in other types of cancer, their use proved that it was possible to obtain a high rate of encapsulation (greater than 90%) and a drug loading greater than 10% and, in some cases, greater than 20% [75,79,80,81,82]. A significant reduction in cell viability in vitro and a significant reduction in tumor volume in in vivo experiments were also proved [75,79,80,81,82]. These data support the fact that the application of SLNs and NCLs in anticancer therapies against cervical cancer may be of special interest and deserves to be researched.

#### 3.1.3. Emulsions and Nanoemulsions

Additionally, to these two types of delivery systems, there is also the group of emulsions and nanoemulsions. This class benefits from the interaction between water and oils through the addition of an emulsifier such as polyglyceryl-10 laurate or PEG 660-stearate to form systems that contribute to flavonoid solubilization and increase their bioavailability [48,83]. Although both emulsions and nanoemulsions have the disadvantage of not being thermodynamically stable systems, dissociating over time, their application results in a high rate of flavonoids encapsulation; normally, greater than 80% decreased aggregation while avoiding gravitational separation [48]. Being distinguished by their size, emulsions have sizes greater than 200 nm while nanoemulsions have sizes smaller than 200 nm. Depending on the intended delivery site for the flavonoids, the use of different ratios of emulsifiers can be tested so that systems may present a suitable size range to more easily reach the target site and accumulate therein [70,84].

Nanoemulsions constituted of soybean phosphatidylcholine and cholesterol have been tested in cervical cancer cells and only showed a 10% viability reduction by using a concentration of 200 µg/mL, although they showed low toxicity, potentiating further studies to increase its therapeutic capacity [48]. In other types of cancer, emulsions and nanoemulsions have shown the same physical characteristics in terms of size, encapsulation rate and stability. Despite this, they possess a high anticancer potential, which is demonstrated by the significant decrease in viable cells. For instance, in melanoma cells, a significant reduction for concentrations above 50 µM of drug encapsulated in the emulsion of lecithin, castor oil and PEG 660-stearate was obtained and in human colorectal carcinoma cells, it was possible to record a reduction of viability around 60% for concentrations of 25 µM drug encapsulated in an emulsion of Labrasol^®^/Tween^®^, lecithin and Miglyol^®^ 812 [83,85,86]. Thereby, formulations with new emulsifiers and different ratios can be tested to improve flavonoid encapsulation, potentiate their anticancer effect, and increase therapeutic index. In this way, significant advances can be achieved toward the development of anticancer therapies based on these molecules.

### 3.2. Polymer-Based Nanoparticles

Polymers have been widely used for flavonoid encapsulation. Polymer-delivery systems were the first and most applied/explored vectors. In general, these delivery systems are constituted of nanoparticles and spherical walls with an outer polymer and a core composed of a hydrophobic surfactant that provides high stability, solubility and bioavailability to the vast majority of flavonoids [55,56,87]. In order to obtain a high final concentration, the formation of this type of system makes use of the flavonoids dissolution in an organic compound, normally ethanol, which is later removed by evaporation under vacuum, or by spray or freeze-drying to reduce the systems cytotoxicity [51,70]. The characteristics of these polymer-based nanoparticles can vary greatly according to the type of polymer considered and can be divided according to their nature, thus being distributed in natural, synthetic or inorganic-based polymers [10,69].

#### 3.2.1. Natural Polymers

Systems based on natural polymers, also called biopolymers, form very diversified systems according to the materials used in their composition in the case of biopolymers are proteins and polysaccharides [69,70]. These polymers guarantee high biocompatibility and biodegradability in addition to low cytotoxicity, making their use in in vitro and in vivo assays widely researched [51,52,53]. However, the use of systems made purely on the basis of proteins or polysaccharides is unusual. In many cases, a combination with another type of biopolymer or with a synthetic or inorganic-based polymer is considered [53]. The use of a polysaccharide such as chitosan is very common, appearing in conjugation with a protein, a polysaccharide or another type of polymer. It was found that this approach provides greater biocompatibility, biodegradability and stability to the systems. In addition, chitosan has mucoadhesive properties that contribute to enhanced delivery of systems to specific/mucosal target sites [49,53,88,89]. The conjugation of a biopolymer with another type of polymer is therefore very common and leads to the formation of systems with better performance for both in vitro and in vivo assays. Generally, they present sizes below 200 nm and high stability and controlled drug release, which facilitates delivery to target cells [49,50,53,90]. Natural polymeric systems based on polysaccharides appear as an important way to guarantee greater bioavailability of systems, often applied in conjugation with other polymers or inclusion complexes [91,92,93].

The use of proteins such as BSA (bovine serum albumin), silk fibroin, keratin and gliadin have already been tested on cervical cancer cell lines. Only silk fibroin has been used without any other associated compound, essentially due to the fact that it is a copolymer bearing hydrophobic and hydrophilic blocks, which facilitates the encapsulation of flavonoids and increases their stability [49,50,51,52,53,94]. However, this polymeric system made of silk fibroin showed a relatively large size compared to other systems as well as a relatively high IC_50_ of 250 µg/mL [52]. The use of polysaccharides in cervical cancer was based only on the conjugation of chitosan with another polymer, such as quinoline or gliadin, and the simple use of focoidan, a polysaccharide with chitosan-like properties, which showed a relatively low IC_50_ of 20 µg/mL, despite the considerably high size of 221 nm [49,53]. In addition to the size reduction that occurs due to the conjugation of various types of polymers, this conjugation also leads to a higher encapsulation rate, normally around 80%, which is higher than the encapsulation rate of 21.81% registered in systems consisting solely of one protein [49,50,51,52,53,94].

#### 3.2.2. Synthetic Polymers

Another type of polymer-based nanoparticles results from synthetic polymers where PEG is the most prominent, often used in conjunction with other types of systems to increase flavonoid solubility and allow a better encapsulation rate [56,62]. In PEG-based systems, the rate of encapsulation is generally high, normally above 90%, although they present a low rate of degradation and low compatibility [56,62,95]. However, the reduction of these negative effects can be significantly mitigated through the formation of systems consisting of a mixture of polymers in order to make their use viable in in vitro and in vivo assays [10,56,69].

In vitro and in vivo studies on anticancer therapies against cervical cancer have already been carried out, with formulated PEG systems conjugated with poly lactide-co-glycolide, poly e-caprolactone conjugated with PEG 1000 succinate, and also gelatine modified pluronic systems [54,55,56,62,94]. In addition, other carriers were also tested in which other types of compounds were used in conjugation with PEG or any of its derivatives. Systems where PEG is not the main compound are mentioned in the section pertaining to this type of compound. Cell viability assays show that it is possible to obtain reduced IC_50_ values close to 10 µM using systems consisting only of synthetic polymers, namely made of PEG and poly lactide-co-glycolide, due to the high blood circulation time that these systems achieve (Table 1). They also revealed a high capacity to be conjugated with specific ligands, as is the case of folic acid, which promotes an active targeting of systems towards cancer cells, considering that these cells have a higher number of folic acid receptors compared to healthy ones [56]. In in vivo studies, the use of poly e-caprolactone and PEG 1000 succinate systems show a tumor weight reduction four times greater than the administration of the drug in its free form [54].

#### 3.2.3. Inorganic Polymers

Delivery systems based on inorganic polymers are a class exhibiting larger diversity of applications, namely in targeted drug delivery, tissue repair, hyperthermia and magnetic resonance imaging [61]. This type of carrier is essentially made up of gold, copper and even iron oxide nanoparticles that commonly form delivery systems as nanoparticles or nanotubes [57,58,59,60,61]. Although these systems have a high aggregation capacity, undergo oxidation and display low stability and biocompatibility, their coverage with polymers such as PEG significantly overcome these disadvantages, providing a place for flavonoids and ligands to bind and thus turning these systems viable [61].

Being characterized by having very small sizes compared to other systems, inorganic polymeric vehicles generally have sizes below 50 nm as well as encapsulation rates between 70 and 80% [57,58,59,60,61]. Iron oxide magnetic nanoparticles coated with various polymers, such as BSA, a-cyclodextrin, citric acid, poly citric acid, PEG or 3- aminopropyl triethoxysilane have been explored to transfect HeLa cells [57,61,64]. Cell viability assays varied greatly according to the system used, and an IC_50_ of 10 µg/mL was obtained for the process mediated by nanoparticles based on iron oxide and BSA [94].

Polymeric carriers have been also widely researched for other cancers, and several systems have already been tested to encapsulate flavonoids and target them to cancer cells. Following this, various polymers are highlighted, namely PEG and chitosan, both mentioned above [87,89,90,95,96]. The incorporation of chitosan in the formation of systems and its conjugation with most types of polymers was tested, for instance, the common conjugation with tripolyphosphate and functionalization with PEG or some type of inclusion complex in order to increase the stability and solubility of encapsulated flavonoids [27,88,97,98]. In addition to the compounds already mentioned, poly(lactic acid), poly(lactic-co-glycolic acid) and polycaprolactone have been also studied, and these systems are also normally conjugated with PEG or one of its derivatives [10,87,95,99,100,101,102]. In vitro assays mediated by these systems showed a decrease in cell viability similar to the one recorded in in vitro assays on HeLa cells, indicating a high coverage of this type of system for any cancer cell.

### 3.3. Micelles

Micelles are constituted by amphiphilic molecules and, depending on the polymeric or lipid nature of these molecules, the formulated systems can be classified as polymeric micelles or lipid micelles [69,103,104]. Due to the molecules that make up the micelles, they offer a hydrophobic core with a high capacity to encapsulate therapeutic agents with low solubility and high hydrophobicity [103,104,105]. For that reason, micelles are ideal for the encapsulation of flavonoids, assuring increased stability of flavonoids as well as a suitable and more consistent release profile [103,104,105]. On the other hand, the outer part of micelles, constituted by the hydrophilic zone of molecules, offers high protection and stability, leading to increased bioavailability of the system [103]. In terms of advantages, this type of delivery system is highly favoured by its small size, often below 100 nm, as well as its high thermodynamic stability, a high drug loading capacity, an increased cellular uptake, and an easy large-scale production. However, formulations vary immensely according to the considered ratios of components, and it may be necessary to perform an intensive study until the ideal ratio is revealed [69,103,104].

In vitro experiments on cervical cancer cells demonstrate that the delivery mediated by micelles constituted of chondroitin sulfate and cholesterol led to a 20% viability reduction at a concentration of 200 µg/mL [65]. In addition, these micelles show a relatively low percentage of encapsulation although they present a high percentage of drug loading, 30.6% and 23.4%, respectively [65]. In vitro assays in other types of cancer, mediated by micelles show better results: an IC_50_ of 110 µM and 21.24 µM in breast cancer and lung cancer cells were achieved, respectively [103,104,105,106,107]. Furthermore, these studies also indicated more favourable physical conditions of formed micelles, with sizes smaller than 100 nm and an encapsulation efficiency greater than 80% [103,104,105,106,107]. The performance of micelles in in vivo studies has also been monitored, and a size reduction higher than three times compared to the use of flavonoid in its free form was observed [108]. The application of micelles for flavonoid encapsulation and delivery needs to be further studied before its broad use. The set of properties displayed by these systems must be optimized to increase their performance as a targeted delivery vector and this certainly will have repercussions on their therapeutic potential against cervical cancer.

### 3.4. Inclusion Complexes

Inclusion complexes are defined as delivery systems characterized by having a host molecule with the ability to trap another molecule using non-covalent forces. Flavonoids can be “trapped” into these complexes due to their capacity to establish hydrophobic interactions with them [10,69,70,92]. The main advantage of inclusion complexes is the fact that they have a cone shape open on both sides with a hydrophilic external surface and a hydrophobic internal surface. Flavonoids can thus be “trapped” in the internal cavity which considerably supports the improvement of their solubility, stability and bioavailability [92]. However, inclusion complexes have some disadvantages, namely a limited encapsulation rate for larger flavonoids, such as glycosylated compounds. Furthermore, they generally have a relatively large size (greater than 200 nm) that limits their use for controlled delivery in in vitro and in vivo studies [91,93,109,110,111]. Following this, due to its low versatility, and the fact that there are cheaper methods of flavonoid encapsulation, its use is somewhat conditioned.

This group of delivery systems is essentially made up of cyclodextrins, of which β-cyclodextrins are the most common. They can be modified in order to change their physical and chemical properties to form more suitable systems according to the site of delivery [92,109,110]. β-cyclodextrins can change their characteristics through chemical modifications so they have more negative or positive charges or a greater or lesser degree of replacement [92,109,110]. There are several types of β-cyclodextrins, with β-cyclodextrin, carboxymethyl-β-cyclodextrin, sulfobutyl ether-β-cyclodextrin and hydroxypropyl-β-cyclodextrin being the most scientifically employed [92,93,109,111,112]. Cyclodextrins can be conjugated with polymers, such as chitosan, to increase their stability, reduce their size and increase their bioavailability [92,93,112]. In addition, conjugation with biotin is also very common due to its receptors being highly expressed in cancer cells, which may contribute to efficiently targeting cancer cells [111]. Other types of delivery systems that are sometimes tested for flavonoid encapsulation are α-cyclodextrins, γ-cyclodextrins and β-lactoglobulins [57,113,114].

In vitro studies on HeLa cells show a viability reduction to 11.5% after an incubation period of 48 h, using inclusion complexes constituted of β-cyclodextrin with a chrysin concentration of 100 µM [67]. In other studies related to other types of cancer, a great reduction of cell viability compared to the use of flavonoids in the free form has also been observed [67,109,111,115]. Therefore, although it is necessary to evaluate the disadvantages already mentioned, the use of inclusion complexes for flavonoids loading/encapsulation and delivery can be considered and investigated since they easily promote flavonoids solubilization in aqueous solutions, where most of them cannot be dissolved.

### 3.5. Other Types of Delivery Systems

Other delivery systems may also emerge as an alternative for the encapsulation and targeting of flavonoids to cancer cells. For instance, dendrimers appear as one of the most promising vehicles.

Dendrimers are a group made up of polymeric materials that have a highly branched architecture with numerous functional groups and an interior cavity that allows the encapsulation of drugs, such as flavonoids [61,116]. Poly(amidoamine) dendrimers are the main and most common type of dendrimers studied, having already been used in in vitro assays in numerous types of cancer and showing significant results, namely a reduction of viability in HeLa cells to 40% using baicalin flavonoid concentration of 25 µg/mL [61,68].

However, their application still has some limitations due to toxicity concerns. Approaches such as PEG conjugation have been considered to minimize this effect and promote a better association with specific ligands [116,117]. In the future, new strategies still need to be investigated to ensure increased stability and bioavailability of these systems to improve their effect on flavonoids encapsulation and subsequent delivery to target cells.

## 4. Conclusions and Future Perspectives

Cancer is still one of the leading causes of death, despite the progress in cancer research and emergent therapies. For instance, currently, there are still no therapies for the effective treatment of cervical cancer, and therefore it is of special importance to develop new methods for enhanced therapeutic effect. In line with this, novel therapies are under development aiming to offer efficient, less invasive, and inexpensive solutions for cancer treatment.

The use of flavonoids as a cancer therapeutic agent has been widely researched in in vitro and in vivo studies. In the cervical cancer context, their application has led to a significant decrease in cell viability and/or tumor growth, despite the low solubility and low bioavailability, as well as easy degradation in acidic medium.

Therefore, delivery methods are currently under development with the goal to create systems that allow the use of lower concentrations of flavonoids and ensure a suitable stability, solubility, bioavailability and delivery profile to cancer cells. To accomplish this, different methodologies have been addressed such as the use of flavonoids encapsulation systems, which can be conjugated to other materials to improve their properties and functionalized with specific ligands such as folic acid to confer them target skills.

In both in vivo and in vitro studies, several types of delivery systems have been tested in therapies against cancer. A variety of delivery systems with high stability promoting targeted and controlled release of flavonoids in cancer cells have been developed and optimized, consisting in a relevant contribution to progress towards the design/conception of flavonoids-based carriers. In cell viability assays, most systems showed a significant reduction in cytotoxicity, with the most promising ones being the lipid systems based on NLCs and the polymeric systems consisting of various types of polymers. The main advantage of NLCs is the high rate of flavonoid encapsulation, normally greater than 90%, as well as a drug loading of around 20% and an almost null cytotoxic effect.

Polymeric systems have been intensely studied in cervical cancer therapy. In this context, the conjugation of biopolymers with other types of polymers offers high biocompatibility, a controlled release of flavonoids and a significant reduction in cell viability and/or tumor size. However, research on polymeric systems still has the opportunity to be optimized, namely by exploring other compounds for polymeric conjugation that can offer high flavonoids stability and solubility.

In conclusion, the continued search for new therapeutic approaches for the treatment of cancer is of utmost relevance. In this sense, flavonoid encapsulation appears as a crucial issue to be further explored and improved, in view of significant advances in different materials, conjugated and functionalized, in the development of high-performance flavonoid-delivery systems for cancer therapy. This will deeply instigate the establishment of an effective and inexpensive method to increase the anticancer action of flavonoids, while minimizing its toxicity to healthy cells, resulting in desirable therapeutic outcomes.

## Figures and Tables

**Table 1:** List of delivery systems used in in vitro HeLa cells and in vivo U14 cervical carcinoma.

Type of Delivery System	Flavonoid	Constitution	Characteristics	Type of Study	Experimental Studies	References
Liposomes	Quercetin	Soybean phosphatidylcholine and cholesterol	Size: 143.1 nm EE ^1^: 96.96%	*In vitro* and *in vivo*	IC_50_^2^: 10–50 µM. DR ^3^: 26.5% after 12 h. Tumor decreases by about 50%.	[43]
		PEG ^4^, cholesterol and soybean phosphatidylcholine	Size: 171.3 nm EE: 81.25%	*In vitro* and *in vivo*	Toxicity: 10% IC_50_: 3.033 µM after 48 h. Tumor decreases from 1500 mm^3^ to 500 mm^3^.	[44]
		Egg-phosphatidylcholine, cholesterol and 2-distearoyl-sn-glycero-3-phosphoethanolamine-PEG 2000	Size: 109.79 nm. EE: superior to 80%	*In vitro*	IC_50_: 185 µM, 40 µM and 14 µM were established after 24, 48 and 72 h, respectively.	[45]
		Triglycerides, lecithin, PEG and acid folic	EE: 96.01%	*In vitro*	IC_50_: 13 µM.	[46]
	Baicalein	Soybean phosphatidylcholine and cholesterol	Size: between 166.9 and 194.6 nm ZP ^5^: between −18.23 and −30.73 mV EE: 44.3%	*In vitro*	Inhibition rate of 66.34%.	[47]
Nanoemulsion	Quercetin	Polyglyceryl-10 laurate, polyglycerol-6 monostearate and sucrose esters-11	Size: between 93 nm and 233 nm EE: 84.7%	*In vitro*	VR ^6^ to 90%.	[48]
Biopolymer	Quercetin	Chitosan and quinoline	Size: 174.8 nm EE: 77.2%	*In vitro*	IC_50_: 10–14 ug/mL after 48 h. DR: 69.3%–78.4% after 8 h.	[49]
	Rutin and quercetin	Keratin and sodium dodecyl sulphate	Size: 55 nm ZP: −28.09 mV EE: 86.5%	*In vitro*	85% was released within 30 h. VR up to 80%.	[50]
	Rutin	Fucoidan	Size: 221 nm	*In vitro*	IC_50_: 20 µg/mL	[51]
	Naringenin	Silk fibroin	Size: between 148.4 and 180.1 nm ZP: between −30.5 and −39.1 mV EE: 21.81%	*In vitro*	IC_50_: 250 µg/mL.	[52]
	Hesperidin	Gliadin coated with chitosan	Size: between 226.5 and 321.40 nm ZP: between −2.91 and 21.40 mV EE: between 73.10 and 80.11%	*In vitro*	IC_50_: 16 ug/mL For blank nanoparticles IC_50_ of 159.33 µg/mL	[53]
Synthetic polymer	Genistein	Poly e-caprolactone and PEG 1000 succinate	Size: 181.83 nm ZP: −14.70 mV EE: 95.56%	*In vitro* and *in vivo*	IC_50_: 24.3 ug/mL, 13.6 µg/mL and 5 µg/mL after 24, 48 and 72 h, respectively. *In vivo* studies showed a reduction of tumour weight by about 4 times.	[54]
	Quercetin	Gelatin modified pluronics	Size: between 79.52 and 152.51 nm EE: 93.02%	*In vitro*	IC_50_: 45.83 µM.	[55]
		PEG and poly lactide-co-glycolide	Size: between 143.1 and 153 nm EE: between 97.8% and 99%	*In vitro*	IC_50_: 10 µM.	[56]
Inorganic polymer	Quercetin	Oxide nanoparticles functionalized with citric acid and α-cyclodextrin	Size: between 22.35 and 59.9 nm ZP: between −15.4 and 35.6 mV EE: higher than 75%	*In vitro*	VR: almost zero for nanoparticles with a drug concentration of 100 µg/mL.	[57]
	Phloretin	Gold nanoparticles	Size: 8 and 15 nm ZP: between −31.7 and −38.2 mV	*In vitro*	VR: 12.5% with a concentration of 4 mg/mL.	[58]
	Hesperetin, naringenin and apigenin	Copper complexes		*In vitro*	Inhibitory rate between 20 and 30%.	[59]
Inorganic polymer/biopolymer	Quercetin	Copper nanoclusters with hydroxyapatite	Size: 36.2 nm ZP: −19.3 mV EE: 72%	*In vitro*	IC_50_: 500 µM.	[60]
Inorganic/synthetic polymer	Quercetin	Magnetic nanoparticles coated with poly citric acid and functionalized with folic acid and PEG	Size: between 10 and 49 nm EE: 80.3%	*In vitro*	VR: 25% with 100 µg/mL of quercetin. Toxicity: 0%	[61]
		Halloysites nanotubes functionalized with PEG	ZP: 37.44 mV	*In vitro*	VR: 30% for a drug concentration of 50 µg/mL. Toxicity: 10%.	[62]
	Quercetin and luteolin	Magnetic iron oxide nanoparticles modified with 3-aminopropyl triethoxysilane, folic acid and PEG	Size: between 8 and 20 nm	*In vitro*	VR: 20% and 40% with 100 µg/mL of quercetin and luteolin, respectively.	[63,64]
Micelles	Quercetin	Chondroitin sulfate and cholesterol	Size: between 124 and 237 nm EE: 30.6%	*In vitro*	VR: near to 80% for a drug concentration of 200 µg/mL.	[65]
Inclusion complex	Fisetin	Cyclosophoraose dimers	Showed 2.4 times more solubility of fisetin than β-cyclodextrin	*In vitro*	VR: 29% after an incubation of 24 h with 100 µM of drug.	[66]
	Chrysin	β-cyclodextrin	Size: 458 nm ZP: −38.4 mV EE: 59.1%	*In vitro*	VR: 11.5% after 48 h with 100 µM of drug.	[67]
Dendrimers	Baicalin	Poly amidoamine dendrimers modified with folic acid	Size: between 174.4 and 258.8 nm ZP: between −2.9 mV and −9.3 mV EE: between 53.5 and 91.9%	*In vitro*	VR: 40% after 48 h with 25 µg/mL of baicalin.	[68]

^1^ EE—Encapsulation efficiency (%). ^2^ IC_50_—Half inhibitory drug concentration. ^3^ DR—Drug release. ^4^ PEG—Polyethylene glycol. ^5^ ZP—Zeta potential (mV). ^6^ VR—Viability reduction.

## Data Availability

Not applicable.

## References

[1] World Health Organization (WHO) (2020). GLOBOCAN 2020—The Global Cancer Observatory: Cancer Today—All Cancers.

[2] World Health Organization (WHO) (2020). GLOBOCAN 2020—The Global Cancer Observatory—Cancer Today Low Income.

[3] Almeida A.M., Queiroz J.A., Sousa F., Sousa Â. (2019). Cervical cancer and HPV infection: Ongoing therapeutic research to counteract the action of E6 and E7 oncoproteins. Drug Discov. Today.

[4] World Health Organization (WHO) (2020). GLOBOCAN 2020—The Global Observatory: Cancer Today—Cervix Uteri.

[5] Lee S.-J., Yang A., Wu T.-C., Hung C.-F. (2016). Immunotherapy for human papillomavirus-associated disease and cervical cancer: Review of clinical and translational research. J. Gynecol. Oncol..

[6] Choi Y.J., Park J.S. (2016). Clinical significance of human papillomavirus genotyping. J. Gynecol. Oncol..

[7] Gomes D., Silvestre S., Duarte A.P., Venuti A., Soares C.P., Passarinha L., Sousa Â. (2021). In silico approaches: A way to unveil novel therapeutic drugs for cervical cancer management. Pharmaceuticals.

[8] Franconi R., Massa S., Paolini F., Vici P., Venuti A. (2020). Plant-derived natural compounds in genetic vaccination and therapy for HPV-associated cancers. Cancers.

[9] Clemente-Soto A.F., Salas-Vidal E., Millan-Pacheco C., Sánchez-Carranza J.N., Peralta-Zaragoza O., González-Maya L. (2019). Quercetin induces G2 phase arrest and apoptosis with the activation of p53 in an E6 expression-independent manner in HPV-positive human cervical cancer-derived cells. Mol. Med. Rep..

[10] Yousefi M., Shadnoush M., Sohrabvandi S., Khorshidian N., Mortazavian A.M. (2021). Encapsulation systems for delivery of flavonoids: A review. Biointerface Res. Appl. Chem..

[11] Lesjak M., Beara I., Simin N., Pintać D., Majkić T., Bekvalac K., Orčić D., Mimica-Dukić N. (2018). Antioxidant and anti-inflammatory activities of quercetin and its derivatives. J. Funct. Foods.

[12] Aziz N., Kim M.-Y., Cho J.Y. (2018). Anti-inflammatory effects of luteolin: A review of in vitro, in vivo, and in silico studies. J. Ethnopharmacol..

[13] Orhan D.D., Özçelik B., Özgen S., Ergun F. (2010). Antibacterial, antifungal, and antiviral activities of some flavonoids. Microbiol. Res..

[14] Madunić J., Madunić I.V., Gajski G., Popić J., Garaj-Vrhovac V. (2018). Apigenin: A dietary flavonoid with diverse anticancer properties. Cancer Lett..

[15] Dobrzynska M., Napierala M., Florek E. (2020). Flavonoid nanoparticles: A promising approach for cancer therapy. Biomolecules.

[16] Imran M., Rauf A., Abu-Izneid T., Nadeem M., Shariati M.A., Khan I.A., Imran A., Orhan I.E., Rizwan M., Atif M. (2019). Luteolin, a flavonoid, as an anticancer agent: A review. Biomed. Pharmacother..

[17] Veeramuthu D., Raja W.R.T., Al-Dhabi N.A., Savarimuthu I. (2017). Flavonoids: Anticancer properties. Flavonoids—From Biosynthesis to Human Health.

[18] Ramos S. (2007). Effects of dietary flavonoids on apoptotic pathways related to cancer chemoprevention. J. Nutr. Biochem..

[19] Cahyana Y., Adiyanti T. (2021). Review: Flavonoids as Antidiabetic Agents. Indones. J. Chem..

[20] Najafi M., Tavakol S., Zarrabi A., Ashrafizadeh M. (2020). Dual role of quercetin in enhancing the efficacy of cisplatin in chemotherapy and protection against its side effects: A review. Arch. Physiol. Biochem..

[21] Siddiqui M., Abdellatif B., Zhai K., Liskova A., Kubatka P., Büsselberg D. (2021). Flavonoids alleviate peripheral neuropathy induced by anticancer drugs. Cancers.

[22] Navarro-Hortal M.D., Varela-López A., Romero-Márquez J.M., Rivas-García L., Speranza L., Battino M., Quiles J.L. (2020). Role of flavonoids against adriamycin toxicity. Food Chem. Toxicol..

[23] Gibellini L., Pinti M., Nasi M., Montagna J.P., De Biasi S., Roat E., Bertoncelli L., Cooper E.L., Cossarizza A. (2011). Quercetin and cancer chemoprevention. Evid. Based Complement. Altern. Med..

[24] Seleem D., Pardi V., Murata R.M. (2017). Review of flavonoids: A diverse group of natural compounds with anti-*Candida albicans* activity in vitro. Arch. Oral Biol..

[25] Liskova A., Samec M., Koklesova L., Brockmueller A., Zhai K., Abdellatif B., Siddiqui M., Biringer K., Kudela E., Pec M. (2021). Flavonoids as an effective sensitizer for anti-cancer therapy: Insights into multi-faceted mechanisms and applicability towards individualized patient profiles. EPMA J..

[26] Panche A.N., Diwan A.D., Chandra S.R. (2016). Flavonoids: An overview. J. Nutr. Sci..

[27] Baksi R., Singh D.P., Borse S.P., Rana R., Sharma V., Nivsarkar M. (2018). In vitro and in vivo anticancer efficacy potential of Quercetin loaded polymeric nanoparticles. Biomed. Pharmacother..

[28] Zhao J., Fang Z., Zha Z., Sun Q., Wang H., Sun M., Qiao B. (2019). Quercetin inhibits cell viability, migration and invasion by regulating miR-16/HOXA10 axis in oral cancer. Eur. J. Pharmacol..

[29] Hsiao Y.-C., Kuo W.-H., Chen P.-N., Chang H.-R., Lin T.-H., Yang W.-E., Hsieh Y.-S., Chu S.-C. (2007). Flavanone and 2′-OH flavanone inhibit metastasis of lung cancer cells via down-regulation of proteinases activities and MAPK pathway. Chem. Biol. Interact..

[30] Cayetano-Salazar L., Olea-Flores M., Zuñiga-Eulogio M.D., Weinstein-Oppenheimer C., Fernández-Tilapa G., Mendoza-Catalán M.A., Zacapala-Gómez A.E., Ortiz-Ortiz J., Ortuño-Pineda C., Navarro-Tito N. (2021). Natural isoflavonoids in invasive cancer therapy: From bench to bedside. Phytother. Res..

[31] Miadoková E. (2009). Isoflavonoids—An overview of their biological activities and potential health benefits. Interdiscip. Toxicol..

[32] Heiss C., Keen C.L., Kelm M. (2010). Flavanols and cardiovascular disease prevention. Eur. Heart J..

[33] Ferreira M.K.A., Fontenelle R.O.S., Magalhães F.E.A., Bandeira P.N., De Menezes J.E.S.A., dos Santos H.S. (2018). Chalcones pharmacological potential: A brief review. Rev. Virtual Quim..

[34] Kuber Banoth R., Thatikonda A. (2020). A review on natural chalcones: An update. Int. J. Pharm. Sci. Res..

[35] Kopustinskiene D.M., Jakstas V., Savickas A., Bernatoniene J. (2020). Flavonoids as anticancer agents. Nutrients.

[36] Sinopoli A., Calogero G., Bartolotta A. (2019). Computational aspects of anthocyanidins and anthocyanins: A review. Food Chem..

[37] Lima B.D.S., Shanmugam S., de Souza Siqueira Quintans J., Quintans-Júnior L.J., de Souza Araújo A.A. (2019). Inclusion complex with cyclodextrins enhances the bioavailability of flavonoid compounds: A systematic review. Phytochem. Rev..

[38] Thilakarathna S.H., Vasantha Rupasinghe H.P. (2013). Flavonoid bioavailability and attempts for bioavailability enhancement. Nutrients.

[39] Ding Y., Tong Z., Jin L., Ye B., Zhou J., Sun Z., Yang H., Hong L., Huang F., Wang W. (2022). An NIR discrete metallacycle constructed from perylene bisimide and tetraphenylethylene fluorophores for imaging-guided cancer radio-chemotherapy. Adv. Mater..

[40] Zhou J., Yu G., Yang J., Shi B., Ye B., Wang M., Huang F., Stang P.J. (2020). Polymeric nanoparticles integrated from discrete organoplatinum(II) metallacycle by stepwise post-assembly polymerization for synergistic cancer therapy. Chem. Mater..

[41] Zhou J., Rao L., Yu G., Cook T.R., Chen X., Huang F. (2021). Supramolecular cancer nanotheranostics. Chem. Soc. Rev..

[42] Khan H., Ullah H., Martorell M., Valdes S.E., Belwal T., Tejada S., Sureda A., Kamal M.A. (2021). Flavonoids nanoparticles in cancer: Treatment, prevention and clinical prospects. Semin. Cancer Biol..

[43] Li J., Shi M., Ma B., Niu R., Zhang H., Kun L. (2017). Antitumor activity and safety evaluation of nanaparticle-based delivery of quercetin through intravenous administration in mice. Mater. Sci. Eng. C.

[44] Li J., Li Z., Gao Y., Liu S., Li K., Wang S., Gao L., Shi M., Liu Z., Han Z. (2021). Effect of a drug delivery system made of quercetin formulated into PEGylation liposomes on cervical carcinoma in vitro and in vivo. J. Nanomater..

[45] Saraswat A.L., Maher T.J. (2020). Development and optimization of stealth liposomal system for enhanced in vitro cytotoxic effect of quercetin. J. Drug Deliv. Sci. Technol..

[46] Ding B., Chen P., Kong Y., Zhai Y., Pang X., Dou J., Zhai G. (2014). Preparation and evaluation of folate-modified lipid nanocapsules for quercetin delivery. J. Drug Target..

[47] Li K., Zhang H., Gao L., Zhai Y., Shi M., Li J., Xiu C., Cao J., Cheng S., Jiang L. (2016). Preparation and characterization of baicalein-loaded nanoliposomes for antitumor therapy. J. Nanomater..

[48] Ni S., Hu C., Sun R., Zhao G., Xia Q. (2017). Nanoemulsions-based delivery systems for encapsulation of Quercetin: Preparation, characterization, and cytotoxicity studies. J. Food Process Eng..

[49] Rahimi S., Khoee S., Ghandi M. (2019). Preparation and characterization of rod-like chitosan–quinoline nanoparticles as pH-responsive nanocarriers for quercetin delivery. Int. J. Biol. Macromol..

[50] Kunjiappan S., Panneerselvam T., Somasundaram B., Sankaranarayanan M., Chowdhury R., Chowdhury A. (2018). Design, in silico modeling, biodistribution study of rutin and quercetin loaded stable human hair keratin nanoparticles intended for anticancer drug delivery. Biomed. Phys. Eng. Express.

[51] Deepika M.S., Thangam R., Sheena T.S., Sasirekha R., Sivasubramanian S., Babu M.D., Jeganathan K., Thirumurugan R. (2019). A novel rutin-fucoidan complex based phytotherapy for cervical cancer through achieving enhanced bioavailability and cancer cell apoptosis. Biomed. Pharmacother..

[52] Fuster M.G., Carissimi G., Montalbán M.G., Víllora G. (2020). Improving anticancer therapy with naringenin-loaded silk fibroin nanoparticles. Nanomaterials.

[53] Filho I.K., Machado C.S., Diedrich C., Karam T.K., Nakamura C.V., Khalil N.M., Mainardes R.M. (2021). Optimized chitosan-coated gliadin nanoparticles improved the hesperidin cytotoxicity over tumor cells. Braz. Arch. Biol. Technol..

[54] Mei L., Zhang H., Zeng X., Huang L., Wang Z., Liu G., Wu Y., Yang C. (2015). Fabrication of genistein-loaded biodegradable TPGS-b-PCL nanoparticles for improved therapeutic effects in cervical cancer cells. Int. J. Nanomed..

[55] Van Thoai D., Nguyen D.T., Dang L.H., Nguyen N.H., Nguyen V.T., Doan P., Nguyen B.T., Van Thu L., Tung N.N., Quyen T.N. (2020). Lipophilic effect of various pluronic-grafted gelatin copolymers on the quercetin delivery efficiency in these self-assembly nanogels. J. Polym. Res..

[56] Elgogary R.I., Rubio N., Wang J.T.-W., Al-Jamal W.T., Bourgognon M., Kafa H., Naeem M., Klippstein R., Abbate V., Leroux F. (2014). Polyethylene glycol conjugated polymeric nanocapsules for targeted delivery of quercetin to folate-expressing cancer cells in vitro and in vivo. ACS Nano.

[57] Ghafelehbashi R., Yaraki M.T., Saremi L.H., Lajevardi A., Haratian M., Astinchap B., Rashidi A.M., Moradian R. (2020). A pH-responsive citric-acid/α-cyclodextrin-functionalized Fe_3_O_4_ nanoparticles as a nanocarrier for quercetin: An experimental and DFT study. Mater. Sci. Eng. C.

[58] Payne J.N., Badwaik V.D., Waghwani H.K., Moolani H.V., Tockstein S., Thompson D.H., Dakshinamurthy R. (2018). Development of dihydrochalcone-functionalized gold nanoparticles for augmented antineoplastic activity. Int. J. Nanomed..

[59] Tan M., Zhu J., Pan Y., Chen Z., Liang H., Liu H., Wang H. (2009). Synthesis, cytotoxic activity, and DNA binding properties of copper (II) complexes with hesperetin, naringenin, and apigenin. Bioinorg. Chem. Appl..

[60] Simon A.T., Dutta D., Chattopadhyay A., Ghosh S.S. (2021). Quercetin-loaded luminescent hydroxyapatite nanoparticles for theranostic application in monolayer and spheroid cultures of cervical cancer cell line in vitro. ACS Appl. Bio Mater..

[61] Mashhadi Malekzadeh A., Ramazani A., Tabatabaei Rezaei S.J., Niknejad H. (2017). Design and construction of multifunctional hyperbranched polymers coated magnetite nanoparticles for both targeting magnetic resonance imaging and cancer therapy. J. Colloid Interface Sci..

[62] Yamina A.M., Fizir M., Itatahine A., He H., Dramou P. (2018). Preparation of multifunctional PEG-graft-halloysite nanotubes for controlled drug release, tumor cell targeting, and bio-imaging. Colloids Surf. B Biointerfaces.

[63] Akal Z., Alpsoy L., Baykal A. (2016). Biomedical applications of SPION@APTES@PEG-folic acid@carboxylated quercetin nanodrug on various cancer cells. Appl. Surf. Sci..

[64] Alpsoy L., Baykal A., Kurtan U., Ülker Z. (2017). Synthesis and characterization of carboxylated luteolin (CL)-functionalized SPION. J. Supercond. Nov. Magn..

[65] Yu C., Gao C., Lü S., Chen C., Huang Y., Liu M. (2013). Redox-responsive shell-sheddable micelles self-assembled from amphiphilic chondroitin sulfate-cholesterol conjugates for triggered intracellular drug release. Chem. Eng. J..

[66] Jeong D., Choi J.M., Choi Y., Jeong K., Cho E., Jung S. (2013). Complexation of fisetin with novel cyclosophoroase dimer to improve solubility and bioavailability. Carbohydr. Polym..

[67] Sundararajan M., Thomas P.A., Venkadeswaran K., Jeganathan K., Geraldine P. (2017). Synthesis and characterization of chrysin-loaded *β*-cyclodextrin-based nanosponges to enhance in-vitro solubility, photostability, drug release, antioxidant effects and antitumorous efficacy. J. Nanosci. Nanotechnol..

[68] Lv T., Yu T., Fang Y., Zhang S., Jiang M., Zhang H., Zhang Y., Li Z., Chen H., Gao Y. (2017). Role of generation on folic acid-modified poly(amidoamine) dendrimers for targeted delivery of baicalin to cancer cells. Mater. Sci. Eng. C.

[69] Wang W., Sun C., Mao L., Ma P., Liu F., Yang J., Gao Y. (2016). The biological activities, chemical stability, metabolism and delivery systems of quercetin: A review. Trends Food Sci. Technol..

[70] Caballero S., Li Y.O., McClements D.J., Davidov-Pardo G. (2021). Encapsulation and delivery of bioactive citrus pomace polyphenols: A review. Crit. Rev. Food Sci. Nutr..

[71] Katopodi A., Detsi A. (2021). Solid lipid nanoparticles and nanostructured lipid carriers of natural products as promising systems for their bioactivity enhancement: The case of essential oils and flavonoids. Colloids Surf. A Physicochem. Eng. Asp..

[72] Patel G., Thakur N.S., Kushwah V., Patil M.D., Nile S.H., Jain S., Banerjee U.C., Kai G. (2020). Liposomal delivery of mycophenolic acid with quercetin for improved breast cancer therapy in SD rats. Front. Bioeng. Biotechnol..

[73] Hu J., Wang J., Wang G., Yao Z., Dang X. (2016). Pharmacokinetics and antitumor efficacy of DSPE-PEG2000 polymeric liposomes loaded with quercetin and temozolomide: Analysis of their effectiveness in enhancing the chemosensitization of drug-resistant glioma cells. Int. J. Mol. Med..

[74] Rezaei-Sadabady R., Eidi A., Zarghami N., Barzegar A. (2016). Intracellular ROS protection efficiency and free radical-scavenging activity of quercetin and quercetin-encapsulated liposomes. Artif. Cells Nanomed. Biotechnol..

[75] Bazylińska U., Pucek A., Sowa M., Matczak-Jon E., Wilk K.A. (2014). Engineering of phosphatidylcholine-based solid lipid nanocarriers for flavonoids delivery. Colloids Surf. A Physicochem. Eng. Asp..

[76] Fathi M., Varshosaz J., Mohebbi M., Shahidi F. (2013). Hesperetin-loaded solid lipid nanoparticles and nanostructure lipid carriers for food fortification: Preparation, characterization, and modeling. Food Bioprocess Technol..

[77] Bose S., Du Y., Takhistov P., Michniak-Kohn B. (2013). Formulation optimization and topical delivery of quercetin from solid lipid based nanosystems. Int. J. Pharm..

[78] Scalia S., Haghi M., Losi V., Trotta V., Young P.M., Traini D. (2013). Quercetin solid lipid microparticles: A flavonoid for inhalation lung delivery. Eur. J. Pharm. Sci..

[79] Sun M., Nie S., Pan X., Zhang R., Fan Z., Wang S. (2014). Quercetin-nanostructured lipid carriers: Characteristics and anti-breast cancer activities in vitro. Colloids Surf. B Biointerfaces.

[80] Liu Y., Zhang H., Cui H., Zhang F., Zhao L., Liu Y., Meng Q. (2022). Combined and targeted drugs delivery system for colorectal cancer treatment: Conatumumab decorated, reactive oxygen species sensitive irinotecan prodrug and quercetin co-loaded nanostructured lipid carriers. Drug Deliv..

[81] Firoozeh N., Mahmoud O., Layasadat K., Mohammadreza A., Esrafil M., Ali K. (2019). Effects of quercetin-loaded nanoparticles on MCF-7 human breast cancer cells. Medicina.

[82] Yostawonkul J., Surassmo S., Iempridee T., Pimtong W., Suktham K., Sajomsang W., Gonil P., Ruktanonchai U.R. (2017). Surface modification of nanostructure lipid carrier (NLC) by oleoyl-quaternized-chitosan as a mucoadhesive nanocarrier. Colloids Surf. B Biointerfaces.

[83] Dora C.L., Silva L.F.C., Mazzarino L., Siqueira J.M., Fernandes D., Pacheco L.K., Maioral M.F., Santos-Silva M.C., Baischl A.L.M., Assreuy J. (2016). Oral delivery of a high quercetin payload nanosized emulsion: In vitro and in vivo activity against B16-F10 melanoma. J. Nanosci. Nanotechnol..

[84] Qian C., McClements D.J. (2011). Formation of nanoemulsions stabilized by model food-grade emulsifiers using high-pressure homogenization: Factors affecting particle size. Food Hydrocoll..

[85] Ting Y., Chiou Y.-S., Pan M.-H., Ho C.-T., Huang Q. (2015). In vitro and in vivo anti-cancer activity of tangeretin against colorectal cancer was enhanced by emulsion-based delivery system. J. Funct. Foods.

[86] Ragelle H., Crauste-Manciet S., Seguin J., Brossard D., Scherman D., Arnaud P., Chabot G.G. (2012). Nanoemulsion formulation of fisetin improves bioavailability and antitumour activity in mice. Int. J. Pharm..

[87] Wang Q., Bao Y., Ahire J., Chao Y. (2013). Co-encapsulation of biodegradable nanoparticles with silicon quantum dots and quercetin for monitored delivery. Adv. Healthcare Mater..

[88] Kim E.S., Kim D.Y., Lee J.-S., Lee H.G. (2021). Quercetin delivery characteristics of chitosan nanoparticles prepared with different molecular weight polyanion cross-linkers. Carbohydr. Polym..

[89] Singh A., Dutta P.K., Kumar H., Kureel A.K., Rai A.K. (2018). Synthesis of chitin-glucan-aldehyde-quercetin conjugate and evaluation of anticancer and antioxidant activities. Carbohydr. Polym..

[90] Pedro R.D.O., Hoffmann S., Pereira S., Goycoolea F.M., Schmitt C.C., Neumann M.G. (2018). Self-assembled amphiphilic chitosan nanoparticles for quercetin delivery to breast cancer cells. Eur. J. Pharm. Biopharm..

[91] Nguyen H.T., Goycoolea F.M. (2017). Chitosan/cyclodextrin/TPP nanoparticles loaded with quercetin as novel bacterial quorum sensing inhibitors. Molecules.

[92] Krauland A.H., Alonso M.J. (2007). Chitosan/cyclodextrin nanoparticles as macromolecular drug delivery system. Int. J. Pharm..

[93] Fülöp Z., Saokham P., Loftsson T. (2014). Sulfobutylether-β-cyclodextrin/chitosan nano- and microparticles and their physicochemical characteristics. Int. J. Pharm..

[94] Zhang X., Xu M., Zhang Z., Hu X., Hao L., Lin Q., Wang S., Jiang W. (2017). Preparation and characterization of magnetic fluorescent microspheres for delivery of kaempferol. Mater. Technol..

[95] Qureshi W.A., Zhao R., Wang H., Ji T., Ding Y., Ihsan A., Mujeeb A., Nie G., Zhao Y. (2016). Co-delivery of doxorubicin and quercetin via mPEG–PLGA copolymer assembly for synergistic anti-tumor efficacy and reducing cardio-toxicity. Sci. Bull..

[96] Liu Y., Liu C., Tang C., Yin C. (2021). Dual stimulus-responsive chitosan-based nanoparticles co-delivering doxorubicin and quercetin for cancer therapy. Mater. Lett..

[97] Konecsni K., Low N.H., Nickerson M.T. (2012). Chitosan–tripolyphosphate submicron particles as the carrier of entrapped rutin. Food Chem..

[98] Bi F., Yong H., Liu J., Zhang X., Shu Y., Liu J. (2020). Development and characterization of chitosan and D-α-tocopheryl polyethylene glycol 1000 succinate composite films containing different flavones. Food Packag. Shelf Life.

[99] Saha C., Kaushik A., Das A., Pal S., Majumder D. (2016). Anthracycline drugs on modified surface of quercetin-loaded polymer nanoparticles: A dual drug delivery model for cancer treatment. PLoS ONE.

[100] Karthick V., Panda S., Kumar V.G., Kumar D., Shrestha L.K., Ariga K., Vasanth K., Chinnathambi S., Dhas T.S., Suganya K.S.U. (2019). Quercetin loaded PLGA microspheres induce apoptosis in breast cancer cells. Appl. Surf. Sci..

[101] Sunoqrot S., Abujamous L. (2019). pH-sensitive polymeric nanoparticles of quercetin as a potential colon cancer-targeted nanomedicine. J. Drug Deliv. Sci. Technol..

[102] Pandey S.K., Patel D.K., Thakur R., Mishra D.P., Maiti P., Haldar C. (2015). Anti-cancer evaluation of quercetin embedded PLA nanoparticles synthesized by emulsified nanoprecipitation. Int. J. Biol. Macromol..

[103] Sheu M.-T., Chen L.-C., Chen Y.-C., Su C.-Y., Hong C.-S., Ho H.-O. (2016). Development and characterization of self-assembling lecithin-based mixed polymeric micelles containing quercetin in cancer treatment and an in vivo pharmacokinetic study. Int. J. Nanomed..

[104] Patra A., Satpathy S., Shenoy A.K., Bush J.A., Kazi M., Hussain M.D. (2018). Formulation and evaluation of mixed polymeric micelles of quercetin for treatment of breast, ovarian, and multidrug resistant cancers. Int. J. Nanomed..

[105] Cote B., Carlson L.J., Rao D.A., Alani A.W.G. (2015). Combinatorial resveratrol and quercetin polymeric micelles mitigate doxorubicin induced cardiotoxicity in vitro and in vivo. J. Control. Release.

[106] Zhao M.-H., Yuan L., Meng L.-Y., Qiu J.-L., Wang C.-B. (2017). Quercetin-loaded mixed micelles exhibit enhanced cytotoxic efficacy in non-small cell lung cancer in vitro. Exp. Ther. Med..

[107] Khonkarn R., Mankhetkorn S., Hennink W.E., Okonogi S. (2011). PEG-OCL micelles for quercetin solubilization and inhibition of cancer cell growth. Eur. J. Pharm. Biopharm..

[108] Gao X., Huang N., Shi H., Ren L., Xu G., Gou H., Gong D. (2015). Enhancing the anti-colon cancer activity of quercetin by self-assembled micelles. Int. J. Nanomed..

[109] Trapani A., Lopedota A., Franco M., Cioffi N., Ieva E., Garcia-Fuentes M., Alonso M.J. (2010). A comparative study of chitosan and chitosan/cyclodextrin nanoparticles as potential carriers for the oral delivery of small peptides. Eur. J. Pharm. Biopharm..

[110] Trapani A., Garcia-Fuentes M., Alonso M.J. (2008). Novel drug nanocarriers combining hydrophilic cyclodextrins and chitosan. Nanotechnology.

[111] Zhu P., Chen L., Zhao Y., Gao C., Yang J., Liao X., Liu D., Yang B. (2021). A novel host-guest complex based on biotin functionalized polyamine-β-cyclodextrin for tumor targeted delivery of luteolin. J. Mol. Struct..

[112] Liu B., Li W., Zhao J., Liu Y., Zhu X., Liang G. (2013). Physicochemical characterisation of the supramolecular structure of luteolin/cyclodextrin inclusion complex. Food Chem..

[113] D’Onofre Couto B., Novaes da Costa R., Castro Laurindo W., Moraes da Silva H., Rocha da Silva C., Sélia dos Reis Coimbra J., Barbosa Mageste A., de Cássia Dias S., José Boggione Santos I. (2021). Characterization, techno-functional properties, and encapsulation efficiency of self-assembled β-lactoglobulin nanostructures. Food Chem..

[114] Zu Y., Wu W., Zhao X., Li Y., Zhong C., Zhang Y. (2014). The high-water solubility of inclusion complex of taxifolin-γ-CD prepared and characterized by the emulsion solvent evaporation and the freeze drying combination method. Int. J. Pharm..

[115] Liu M., Liao R., Zhao Y., Yang B. (2015). Host—Guest Inclusion System of Luteolin with Polyamine-β-cyclodextrin: Preparation, Characterisation, Anti-oxidant and Anti-cancer Activity. Aust. J. Chem..

[116] Luong D., Kesharwani P., Deshmukh R., Mohd Amin M.C.I., Gupta U., Greish K., Iyer A.K. (2016). PEGylated PAMAM dendrimers: Enhancing efficacy and mitigating toxicity for effective anticancer drug and gene delivery. Acta Biomater..

[117] Chauhan A.S. (2018). Dendrimers for drug delivery. Molecules.

